# Association between preterm birth and thoracic musculoskeletal static alterations in adolescents

**DOI:** 10.1590/bjpt-rbf.2014.0074

**Published:** 2015

**Authors:** Kessey M. B. Garcia, Josy Davidson, Ana L. Goulart, Amelia M. N. dos Santos

**Affiliations:** Departamento de Pediatria, Universidade Federal de São Paulo (UNIFESP), São Paulo, SP, Brazil

**Keywords:** physical therapy, adolescent, prematurity, thorax, posture, photogrammetry

## Abstract

**OBJECTIVE:**

To compare thoracic musculoskeletal static alterations in adolescents born prematurely with those born at term and investigate neonatal and post-neonatal variables associated with thoracic alterations.

**METHOD:**

This is a cross-sectional study with 57 adolescents aged 10-15 years born prematurely and 57 adolescents born at term paired by gender and age. Photographs of the head and thorax in the front, back, and right side views were studied using a computer program. The two groups were compared in regards to: elevation of clavicles, elevation of shoulders, protrusion of the head, and anteroposterior and mediolateral thoracic length. Factor associated with thoracic disorders were evaluated by linear regression analysis.

**RESULTS:**

The Preterm group had mean gestational age of 32.0±2.8 weeks and the birth weight was 1462±338 and 3342±430 g for the Preterm and Term adolescents, respectively. Preterm adolescents had higher elevation of the left shoulder (22.7±5.4^o^ vs. 20.6±5.3^o^;sim, p=0.038) and the right shoulder (22.2±4.4^o^ vs. 18.5±5.7^o^; p<0.001). Smaller protrusion of the head (27.8±6.1^o^ vs. 32.4±7.9^o^; p=0.008), mediolateral thoracic length (22.9±2.3 cm vs. 25.1±3.1 cm; p<0.001) and anteroposterior thoracic length (19.7±2.2 cm vs. 21.1±3.4 cm; p<0.001) were found in preterm adolescents. By multiple regression analysis, factors associated with higher shoulder elevation were birth weight <1500 g (p<0.001) and mechanical ventilation during neonatal period >5 days (p=0.009).

**CONCLUSION:**

Adolescents born prematurely presented greater thoracic musculoskeletal static alterations compared to those born at term. Factors associated with these alterations were: very low birth weight and longer duration of mechanical ventilation in the neonatal unit.

## Introduction

As the survival rate of preterm newborns has increased, so has neonatal and postnatal morbidity^1^^,^ 2, requiring a long-term follow up and continuous evaluation of health problems stemming from premature birth, such as respiratory disorders that can persist into childhood, adolescence, and possibly even adulthood^3^^,^ 4. These alterations could compromise functional capacity and biomechanical conditions, worsening lung function and rib cage structure.

A study with children aged 6-9 years born prematurely with very low birth weight compared with children born at full term, paired by sex and age, showed that children born prematurely walked shorter distances during the six-minute walking test. By linear multiple regression analysis, these authors found that bronchopulmonary dysplasia had a negative influence on the walking distance during the test^5^.

Similar to what is seen in patients with chronic lung disease^6^^-^ 8, it is possible that respiratory disorders and mechanical ventilation in the neonatal period may lead to long-term alterations in the thoracic cage. In infants born prematurely, thoracic alterations were found in more than 65% of 121 infants born at 31.1±2.8 weeks of gestation in the first year of age^9^. In that study, 40.5% of infants born prematurely had shoulder elevation, 45.5% presented costal retraction, and 14.0% had both thoracic alterations. Those authors showed that factors associated with these thoracic alterations were respiratory distress syndrome in the neonatal period (OR: 3.2; 95% CI: 1.2-8.9), need for oxygen therapy at 28 days of life (OR: 11.1; 95% CI: 1.3-92.6), and low index of height/age (OR: 4.6; 95% CI: 1.3-15.2), showing the influence of the neonatal respiratory condition on musculoskeletal static thoracic characteristics at one year of age^9^. In the same way, Barros^10^ studied 28 infants born prematurely in their first year of life and found thoracic deformities in 36% and shoulder elevation or costal retractions in 68% of the studied infants.

These findings raise questions in regard to the persistence of such alterations at older age. However, there are no studies addressing alterations in the rib cage in adolescents stemming from premature birth.

In this context, the aim of the present study was to compare static musculoskeletal characteristics of the thoracic cage in adolescents born prematurely with those born at full term and to evaluate neonatal and post-neonatal variables associated with these thoracic disorders.

## Method

A cross-sectional study was conducted involving two groups of adolescents aged 10 to 15 years, matched by age and gender (1:1). The inclusion criteria for the Preterm group were adolescents born with a gestational age <37 weeks and a birth weight <2000 g, followed up at the Multi Disciplinary Premature Outpatient Clinic of a public university. The inclusion criteria for the Term group were healthy adolescents born at term with birth weight >2500 g, who were discharged from the maternity within three days of life; who neither presented any clinical complication nor required oxygen therapy in the neonatal period, had not been hospitalized after discharge from the maternity until the day they were included in the study, and were attending a public school.

The exclusion criteria were congenital malformation, neuromuscular disease, chromosome disorder, any orthopedic anatomical defect which could interfere with static posture or severe neurological impairment based on magnetic resonance or computed tomography imaging performed in the neonatal period and/or during follow up and a neurologic evaluation performed by a pediatric neurologist during follow up at the Premature Outpatient Clinic.

This study was approved by the Ethics Committee of *Universidade Federal de São Paulo* (UNIFESP), São Paulo, SP, Brazil (#1830-07), and parents/guardians signed an informed consent form.

The following demographic and clinical data were collected from the Term group: gender, birth weight, gestational age, and duration of stay in the maternity at the time of birth. Besides these data, the following were also collected from the Preterm group: gestational age based on the best obstetric estimate or pediatric assessment^11^, Apgar score, clinical complications in the neonatal unit, duration of mechanical ventilation and duration of hospitalization in the neonatal unit. Upon enrollment in the study, the following data were collected from both groups: chronological age, weight, stature, and body mass index^12^.

### Photography

Photographs of the head and thorax in the front, back, and right side views were taken using a digital camera (Sony Cyber-shot(r) DSC-T10) placed on a tripod at a distance of three meters from the participant and one meter from the floor. The adolescents wore bathing suits for the visualization of the anatomic points marked with half-moon polystyrene disks placed on the acromia, manubrium, and trapezius muscles, based on Davidson et al.^9^, as well as the ear lobes, seventh cervical vertebra (C7), third thoracic vertebra (T3), and inferior angle of the scapulae, based on Ferreira et al.^13^.

### Image analysis

**Figure 1:**
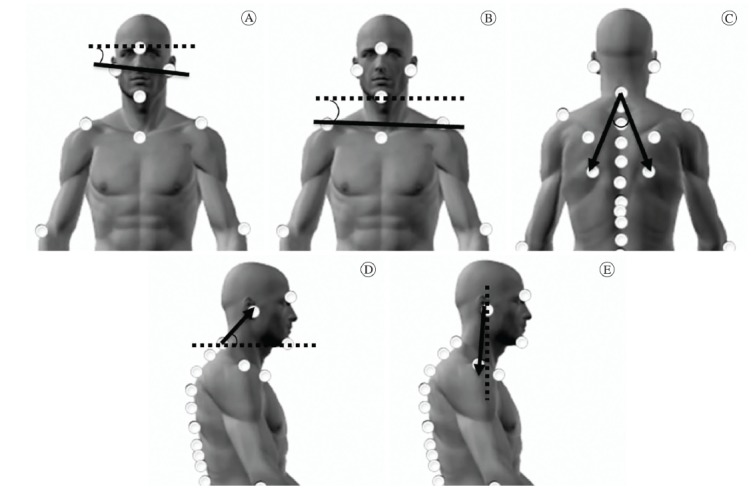
Representation of posture measures; inclination of head (1A); inclination of shoulders (1B); asymmetry of scapulae (1C); forward lean of head (1D); alignment of head in relation to shoulder (1E).

**Figure 2:**
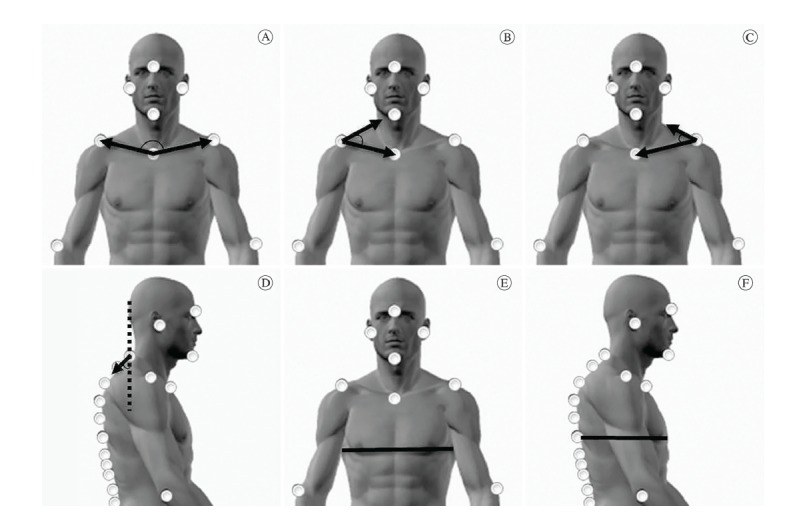
Representation of measures for evaluation of thoracic alterations; elevation of clavicles (2A); elevation of right and left shoulder (2B and 2C); protrusion of head (2D); mediolateral thoracic distance (2E); anteroposterior thoracic distance (2F).

Postural measures (Figure 1) were evaluated in order to control possible interference from the body position on the conformation of the thoracic cage during photography. The following postural measures were evaluated by photogrammetry by a previously trained physical therapist^9^ based on recommendations by Ferreira et al.^13^:

- Inclination of the head (1A): angle between both ear lobes and line parallel to the ground;
- Inclination of the shoulders (1B): angle between the acromia and line parallel to the ground;
- Asymmetry of scapulae (1C): difference in distance between right and left scapula and C7;
- Forward lean of head (1D): angle between C7, ear lobe, and line parallel to the ground;
- Alignment of head in relation to shoulder (1E): angle between acromion, ear lobe, and line perpendicular to the ground;

For the evaluation of the angles and measures related to thoracic alterations, the following were analyzed (Figure 2):

- Elevation of clavicles (2A): angle between the acromia and manubrium^9^;
- Elevation of shoulders (2B and 2C): angle between acromion, trapezius, and manubrium (right and left)^9^;
- Protrusion of head (2D): angle between C7, T3, and a line perpendicular to the ground^13^;
- Thorax dimensions (2E and 2F): mediolateral and anteroposterior distance of the thorax^13^.

A postural assessment software program (SAPO) was used for the analysis of the images, obeying the following sequence: opening of file, plumb line calibration of image, 100% zoom, marking of desired anatomic points and release of report. Besides the angles related to the thorax, the thoracic distances formed between the dermis extremities on the mammary line in the front view (2E) and side view (2F) were calculated using the software tool, Edge Detection^13^. This software has been reported as a reliable method with good reproducibility and intra- and inter-rater agreement for most angle measures in adults and children^9^^,^ 14 ^-^ 16.

### Statistical analysis

Convenience sampling was performed with the inclusion of all adolescents born prematurely and followed at the Premature Outpatient Clinic of the institution. The numerical variables were expressed as mean and standard deviation or median, range, Q1-Q3 (first and third quartile) and compared using either the Student t-test (normal distribution) or the Mann-Whitney test (non-normal distribution). The categorical variables were expressed as frequency and compared using either the chi-squared (χ^2^) test or Fisher's exact test. Factors associated with thoracic alterations were studied by univariate linear regression analysis, considering clinical variables that may have interfered with these alterations. Then, variables with clinical interest and a statistical significance level of p<0.2 in the univariate analysis were included in the multiple linear regression model.

The sample power for the evaluation of the angles and measures related to thoracic alterations was calculated after data analysis.

The statistical analyses were conducted using SPSS for Win/v.17.0 (IBM SPSS Statistics, Somers, NY, USA). P<0.05 was considered statistically significant.

## Results

During the study period, 76 adolescents aged 10 to 15 years were monitored at the Premature Outpatient Clinic. Nine (11.8%) subjects were excluded from the study; 8 (10.5%) due to neurological sequel from intra-ventricular hemorrhage, periventricular leukomalacia, epilepsy or chromosome disorder, and 1 (1.3%) due to differences in the length of the lower limbs. Among the 67 eligible adolescents, 10 (14.9%) were not included [4 (6.0%) due to refusals on the part of parents/guardians and 6 (9.0%) because matching was not possible]. Thus, 57 (85.1%) adolescents in the Preterm group were matched by age and gender with 57 adolescents born at full term.

Adolescents born at term had gestational age >37 weeks, mean birth weight of 3342±430 g (median: 3400 g; range: 2585 - 4000 g; Q1 [1^st^ quartile - Q3: 3^rd^ quartile: 2995-3750 g). None of them presented any clinical complication nor required oxygen therapy in the neonatal period and were discharged from the neonatal unit within three days of age.

The premature group had a lower birth weight than the control group (1462±338 g vs. 3342±430 g; p<0.001). In the premature group, mean gestational age was 32.0±2.8 weeks (median: 32.0; range: 26.9 to 36.6 weeks; Q1- Q3: 29.3-33.9), mean Apgar score at first minute was 7±2 (median: 7; range: 1 to 9; Q1-Q3: 6-8) and mean Apgar score at fifth minute was 9±1 (median: 9; range: 6 to 10; Q1-Q3: 9-9).

Regarding clinical status in the neonatal period, 17 (29.8%) participants in the premature group experienced respiratory distress syndrome, 6 (10.5%) developed neonatal sepsis, 1 (1.8%) developed meningitis, 18 (31.6%) intraventricular hemorrhage, 6 (10.5%) were oxygen dependent at 28 days of life, and 2 (3.5%) were oxygen dependent at 36 weeks of corrected gestational age. During hospitalization in the neonatal unit, 19 (33.3%) preterm children were submitted to mechanical ventilation for a mean of 2±7 days (median: zero; range: 0 to 48 days; Q1-Q3: 0-1). Mean of days of hospital stay in the neonatal unit for those born prematurely was 37±22 days (median: 33; range: 10-130 days; Q1-Q3: 20-48 days).

At study entry, the Preterm group was similar to the control group with regard to age (11.5±1.3 vs. 11.4±1.3 years; p=0.949), weight (39.2±9.8 vs. 39.3±7.1 Kg; p=0.946), height (145.5±10.7 vs. 146.5±9.3 cm; p=0.586), body mass index (18.3±3.3 vs. 18.3±2.6 Kg/cm^2^; p=0.916), percentage of obese adolescents (3.5 vs. 0%; p=0.496), percentage of patients with current asthma (5.3 vs. 7.0%; p=0.717), and percentage of adolescents with physical activities for at least twice/week (98.5% vs. 100%; p=0.500). Fifty-four adolescents in the Preterm group (94.7%) were right-handed and 3 (5.3%) were left-handed, whereas 54 (94.7%) adolescents in the Term group were right-handed, 2 (3.5%) were left-handed and 1 (1.8%) was ambidextrous (p=0.463).

The measures related to posture in the cervical and thoracic region were similar in both groups, with the exception of the alignment of the head in relation to the shoulders (Table 1). The measures of angles and those related to thoracic alterations, including sample power, are shown in Table 2. Statistically significant differences were found between groups in the angular and linear measures related to the thoracic cage, with the exception of the angle between the acromia and manubrium (elevation of clavicles) and the ratio between the mediolateral and anteroposterior thoracic distances (Table 2).

**Table 1:** Analysis of posture measures of the two groups of adolescents.

	Preterm Group (n=57)	Term Group(n=57)	p	Sample power
Inclination of the head (degrees)	2.7±2.1	2.3±1.7	0.236^#^	<65%
Inclination of the shoulder (degrees)	1.5±1.3	1.7±1.2	0.460^#^	<65%
Asymmetry of the scapula (degrees)	17.3±12.3	15.3±12.7	0.400^#^	<65%
Anterior inclination of the head (degrees)	45.7±6.1	44.9±10.4	0.631^#^	<65%
Head alignment in relation to shoulder (degrees)	9.4±6.1	12.6±9.2	0.028^#^	85%

# p: Mann-Whitney test.

**Table 2:** Analysis of thoracic measures of adolescents included in study.

	Preterm Group(n=57)	Term Group (n=57)	p	Sample power
Elevation of clavicles (degrees)	172.8±7.0	175.3±8.0	0.075^#^	<65%
Elevation of right shoulder (degrees)	22.4±4.4	18.5±5.7	<0.001^#^	95%
Elevation of left shoulder (degrees)	22.7±5.4	20.6±5.3	0.038^#^	70%
Protrusion of head (degrees)	27.8±6.1	32.4±7.9	0.001^#^	95%
Mediolateral thoracic lenght (A) (cm)	22.9±2.3	25.1±3.1	0.008^#^	95%
Anteroposterior thoracic lenght (B) (cm)	19.7±2.2	21.1±3.4	<0.000^#^	95%
A/B ratio	1.2±0.1	1.2±0.1	0.085^&^	95%

# p: Mann-Whitney test;

& p: t-test.

Univariate linear regression analysis showed that shoulder elevation, head protrusion and thorax size thoracic were associated with gestational age <37 weeks. In adolescents born prematurely, compared to those born at term, the following differences were found by the univariate linear regression: the angle of head protrusion was -4.649° (95% CI: -7,30 to -2,00; p=0.001) lower; the mediolateral thoracic length was -1.46 cm (95% CI: -2.74 to -0.40; p=0.007) shorter; and the anteroposterior thoracic length was -2.16 cm (95% CI: -3.16 to -1.16; p<0.001) shorter. Moreover, the degree of shoulder elevation was positively associated with birth weight <1500 grams, gestational age <37 weeks, need for mechanical ventilation for more than five days, and need for oxygen therapy for more than 10 days (Table 3).

**Table 3:** Univariate and multivariate linear regression analysis for factors associated with shoulder elevation (degree).

	Beta	95% CI	p
**Univariate linear regression analysis**			
Birth weight < 1500 grams	5.147^o^	2.976-7.319	<0.001
Gestational age < 37 weeks	3.668^o^	1.769-5.568	<0.001
Need for O_2_ therapy > 28 days of life	1.440^o^	-2.421-5.381	0.458
Need for mechanical ventilation > 5 days	7.593^o^	3.299-11.886	0.001
Oxygen therapy for > 10 days	5.082^o^	1.641-8.523	0.004
Current wheezing	1.192^o^	-3.009-5.393	0.575
Overweight/obesity	0.221^o^	-2.154-2.596	0.854
**Multivariate linear regression analysis**			
Birth weight < 1500 grams	4.45^o^	2.27-6.63	<0.001
Need for mechanical ventilation > 5 days	5.57^o^	1.43-9.72	0.009

O_2_: Oxygen.

By multivariate linear regression analysis, after adjustment for variables with level of p value <0.2, gestational age <37 weeks was significantly associated with lower head protrusion, smaller thorax size and shoulder elevation. Moreover, the shoulder elevation was also found to be higher in adolescents with birth weight <1500 g and those submitted to mechanical ventilation >5 days during neonatal period, compared to those without these characteristics (Table 3).

## Discussion

To our knowledge, this is the first study to assess thoracic alterations in adolescents born prematurely. This study showed that such adolescents exhibit higher shoulder elevation, retracted head posture, and smaller thorax compared to adolescents born at term. Moreover, shoulder elevation was positively associated with very low birth weight and need for mechanical ventilation for more than five days in the neonatal unit.

Higher shoulder elevation may be due to increased respiratory effort, which affects the rib cage muscles, with an increase in the activity of the erector spinae, large dorsal, greater pectoral, and trapezius muscles as previously reported in patients with chronic respiratory problems such as asthma, mouth breathers, and chronic obstructive pulmonary diseases^6^^-^ 8 ^,^ 17.

The extension of the cervical vertebrae with lesser head protrusion is believed to occur when there is narrowing of the pharynx. With the extension of the cervical spine, there is the release of the airways, thereby facilitating breathing in individuals with airway obstruction, such as those with asthma and/or mouth breathing^18^^,^ 19. It is possible that the same mechanism may occur in adolescents born prematurely, who presented lesser head protrusion, favoring a retracted head posture.

The higher shoulder elevation and retracted posture found in this study in adolescents born prematurely may be related to patterns of obstructive disorders observed in pulmonary function tests in preterm children and adolescents, mainly in those who also developed bronchopulmonary dysplasia^20^.

The anteroposterior and mediolateral measures of the thorax which were smaller in the Preterm group reflect the smaller size of the thoracic cage and this may be related to lung function given that children and adolescents born prematurely can have smaller lung volumes^21^^,^ 22 that could lead to lesser growth of the thoracic cage. Moreover, the respiratory muscles may become altered over time due to the respiratory muscle work in childhood, causing distortions and lesser development of the thoracic cage in children with respiratory disorders^6^^,^ 7 or in premature children with bronchopulmonary dysplasia^22^^,^ 23. A similar situation may occur in patients with chronic lung conditions, such as asthma or cystic fibrosis, due to the shortening of the thoracic musculature and flattening of the diaphragm^6^^,^ 7.

The mechanics and conformation of the thorax vary depending on gender, age, and body mass. As an integral part of the thoracic structure, the spinal column also influences the conformation of the rib cage. A study involving children and adolescents between eight and 14 years of age found changes in the vertical alignment with age; moreover, greater lordosis was found among females in comparison with males during flexion of the shoulders, which may be attributed to greater muscle weakness in girls in the post-menarche period^24^.

Other studies demonstrated the importance of nutritional status on lung function, which could indirectly affect thoracic conformation. Obese children and adolescents between six and 14 years of age present lesser respiratory muscle strength in comparison with those within the ideal weight range, especially with regard to expiratory capacity, which can compromise ventilatory capacity^25^. This limitation has also been observed in other age groups, demonstrating lesser functional residual capacity in obese adults^26^^-^ 28. Moreover, excess weight and obesity affect posture, balance, and both upper and lower limb strength^29^^,^ 30.

For these reasons, in order to control variables that could interfere with the thoracic frame and size, in this study, the two groups of adolescents were paired by age and gender, and other variables that could alter our results such as weight, height, and body mass index were found to be similar between groups.

Cervical and thoracic postures were also evaluated to verify the positioning of the adolescents during photography. In order to minimize and control the influence of global posture, other measurements of the thorax and head position were analyzed (Table 2). This evaluation demonstrated that measures related to global posture were similar in both groups, except for the alignment of the head in relation to the shoulders, which was reduced in the Preterm group. This finding indicates a retracted head posture in relation to the shoulders in adolescents born prematurely, which may represent a better position to ensure the opening of the airways.

As shown by the adjusted linear regression analysis, this study highlighted the hypothesis that preterm birth, particularly if followed by mechanical ventilation during the neonatal period, may lead to thoracic alterations in adolescence. In this study, after adjustment for covariate variables, very low birth weight and mechanical ventilation for more than had five days were associated with higher shoulder elevation.

It is possible that the thoracic alterations found in the present study are accompanied by lung function impairment stemming from premature birth and associated comorbidities, as previously reported for children and adolescents with chronic lung disease^7^ and premature children with pulmonary dysplasia^9^^,^ 21. Lung tests would allow assessing the association between lung impairment in adolescents with musculoskeletal alterations of the thoracic cage as well as quantifying and determining the type of functional abnormality. A study is currently underway by our research group to assess the association between postural change and impaired lung function in infants born prematurely.

Convenience sampling and a lack of a sample size calculation are the limitations of this study. However, in the post hoc analysis, considering a two-tailed test and 5% alpha error, the sample power of this study was greater than 95% for elevation of the right shoulder, head protrusion, and for thorax size.

While there is no standard of normality for musculoskeletal structures of the thorax, significant differences were found between adolescents born prematurely and those born at full term. It is possible that such differences may be attributed to the premature birth because, in addition to the neonatal characteristics, the adolescents were similar regarding variables that could affect the conformation of the thoracic cage.

This study highlights the influence of the preterm birth over respiratory system development and reinforces the need for long-term follow up of these patients by a multi-professional team in order to search for any thoracic alterations and plan for early interventions. Thus, it can be concluded that adolescents born prematurely presented greater thoracic musculoskeletal static alterations compared to those born at full term and that the factors associated with these alterations were very low birth weight and longer duration of mechanical ventilation in the neonatal unit.
